# Supplementary figures and images for: Longitudinal expression profiling of CD4+ and CD8+ cells in patients with active to quiescent giant cell arteritis
Source: BMC Med Genomics. 2018 Jul 23;11:61. doi: 10.1186/s12920-018-0376-4 (PMC6057030; doi:10.1186/s12920-018-0376-4)

**Supplementary Table 1**.

**
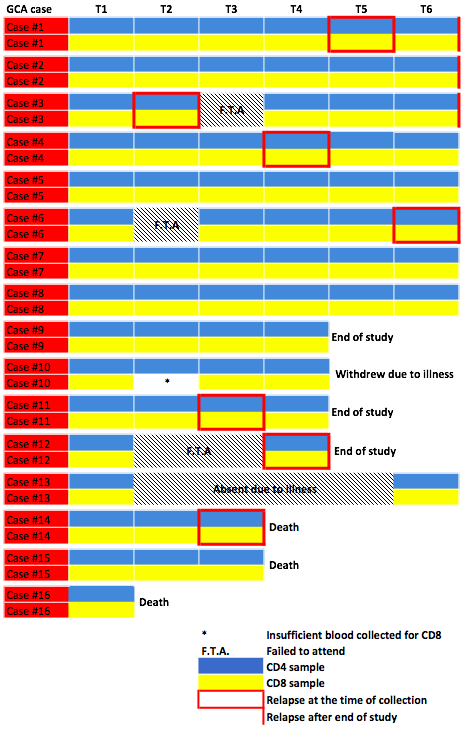
**

Supplement: Supplementary file 1 — Table S1. Cases recruited and attendance for all 6 time points (DOCX 160 kb) [file 12920_2018_376_MOESM1_ESM.docx]

**Supplementary Figure 1.**


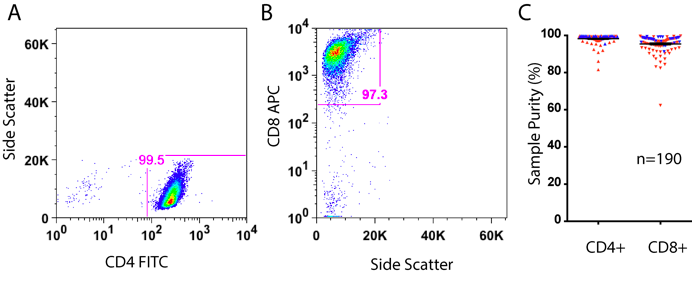

Supplement: Supplementary file 2 — Figure S1. Quality control metrics for stored specimens. Representative FACS analysis for FITC bound CD4 (A) and APC bound CD8 cells (B). Panel C displays the FACS confirmed purity of all specimens, with case and control samples represented by red and blue triangles respectively. (DOCX 100 kb) [file 12920_2018_376_MOESM2_ESM.docx]

**Supplementary Figure 2.**


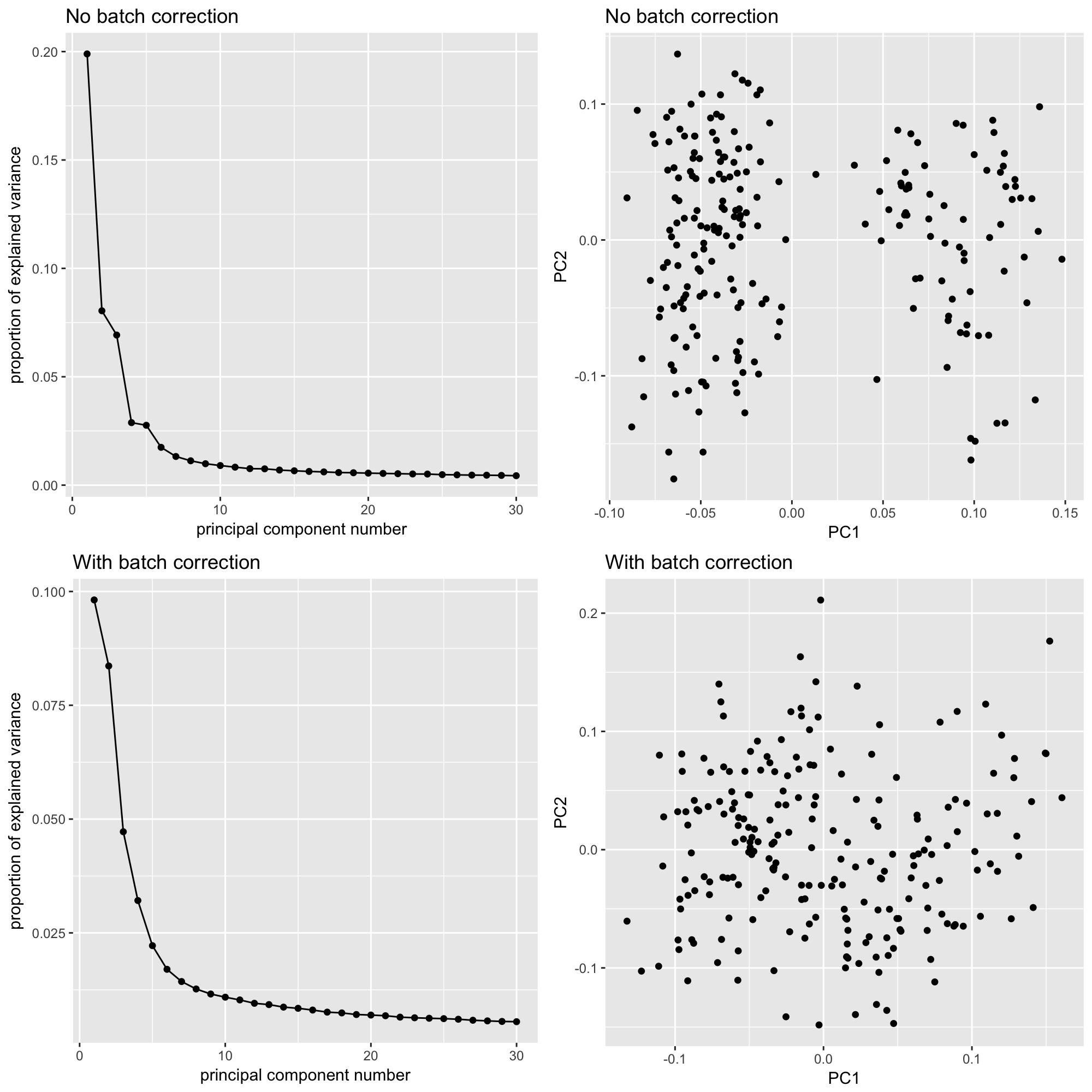

Supplement: Supplementary file 3 — Figure S2. Effect of batch correction on 195 samples (2 samples of the 197 were removed). Three parameters (Flowcell ID, Gender and Ethnicity) were used to remove confounding effects in edgeR. PC1 contributes the greatest amount of variance and is largely attributed to Flowcell ID, which accounts for most of the variance in sequencing experiments. (DOCX 384 kb) [file 12920_2018_376_MOESM3_ESM.docx]

**Supplementary Figure 3.**


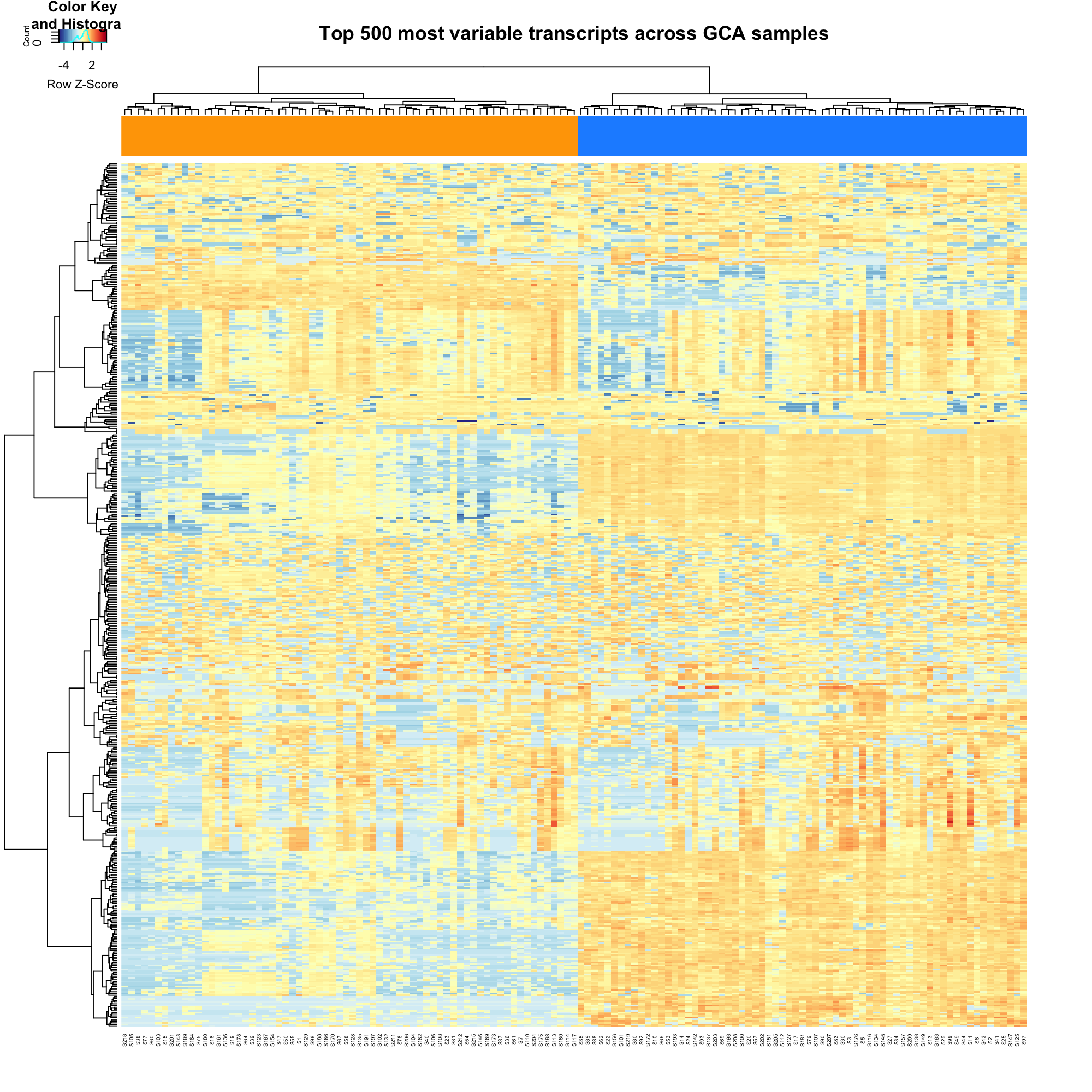

Supplement: Supplementary file 6 — Figure S3. Expression levels of the top 500 most variable transcripts in CD4 and CD8 cells, shown for each of 135 samples. Sample groups are indicated by the orange (CD4) and blue (CD8) bars at the top of the heatmap. (DOCX 555 kb) [file 12920_2018_376_MOESM6_ESM.docx]
