# Supplementary material for: Longitudinal expression profiling of CD4+ and CD8+ cells in patients with active to quiescent giant cell arteritis
Source: BMC Med Genomics. 2018 Jul 23;11:61. doi: 10.1186/s12920-018-0376-4 (PMC6057030; doi:10.1186/s12920-018-0376-4)
Supplement: Supplementary file 4 — Table S2. Ophthalmic clinical summary data (GCA cases, n = 16). (DOCX 51 kb) [file 12920_2018_376_MOESM4_ESM.docx]

**Supplementary Table 2.**

|  | **Number of cases** |
| --- | --- |
| ***Visual disturbance at T1*** | |
| **Monocular** | **10** |
| **Bilateral** | **4** |
| **None** | **2** |
| ***Eye* *affected*** | |
| **Right** | **4** |
| **Left** | **5** |
| **Both** | **4** |
| ***Ophthalmic Manifestation*** | |
| **Amaurosis Fugax** | **2** |
| **AION** | **9** |
| **Transient diplopia** | **1** |
| **3rd Cranial nerve palsy** | **2** |
| ***Final recorded visual outcome*** | |
| **Normal vision both eyes** | **4** |
| **Normal vision one eye, visual impairment other** | **7** |
| **Blind one eye** | **2** |
| **Blind both eyes** | **3** |

Definitions (Snellen Chart): *Normal vision:* >6/9; *Vision impairment:* <6/9 but >6/60; *Severe vision impairment (“Blind”):* <=6/60.
