# Supplementary material for: Longitudinal expression profiling of CD4+ and CD8+ cells in patients with active to quiescent giant cell arteritis
Source: BMC Med Genomics. 2018 Jul 23;11:61. doi: 10.1186/s12920-018-0376-4 (PMC6057030; doi:10.1186/s12920-018-0376-4)
Supplement: Supplementary file 5 — Table S3. General disease outcome and prognostic measures. (DOCX 38 kb) [file 12920_2018_376_MOESM5_ESM.docx]

**Supplementary Table 3.**

| **Category** | **Number of cases** |
| --- | --- |
| ***Number of relapse events during the 12 month study period*** | |
| **None** | **5** |
| **One** | **5** |
| **Two** | **2** |
| **Three or more** | **1** |
| **Unknown (loss to follow up)** | **3** |
| ***Deceased within 12 months*** | |
| **Yes** | **3** |
